# Fatal Viscerocutaneous Brown Recluse Envenomation With Orbital Compartment Syndrome

**DOI:** 10.7759/cureus.60943

**Published:** 2024-05-23

**Authors:** Jonathan W Meadows, Nima Shayesteh, Eric Crandall, Sarah A Watkins

**Affiliations:** 1 Department of Emergency Medicine, Franciscan Health Olympia Fields, Olympia Fields, USA; 2 Department of Emergency Medicine, Del Sol Medical Center, El Paso, USA; 3 Department of Emergency Medicine, Texas Tech University Health Sciences Center, West Texas Regional Poison Center, El Paso, USA

**Keywords:** poison center, brown recluse spider bite, orbital compartment, lateral canthotomy, loxoscelism

## Abstract

*Loxosceles *is an arachnid genus comprising several species in the United States, popularly known as brown recluse spiders. The venom is cytotoxic, complex, and has a mixture of many proteins, some of which function as proteases. Envenomation can cause necrotic skin lesions that may become extensive and take many months to heal. Even more rarely, venom may cause systemic effects, leading to widespread hemolysis, coagulopathy, and death. These symptoms typically occur rapidly within 24-48 hours following the bite. We describe a rare case of a 44-year-old male with fatal systemic loxoscelism with orbital compartment syndrome requiring emergent lateral canthotomy and cantholysis.

## Introduction

In 2021, there were 566 brown recluse spider bite cases mentioned, with one death in the USA [1]. There are 11 species of the genus *Loxosceles* in the USA. Four are in Texas, each with a different geographic distribution [2]. South America’s *L. laeta* has the highest mortality [2]. *Loxosceles* is from the Greek word meaning “crooked or slanted legs” when resting [2]. It has three non-touching pairs of eyes, tan legs, and a classic violin-shaped pattern on the dorsal cephalothorax, noting the “violin or fiddleback spider” feature [2]. There is body and color variation among species, leading to potential misclassification and excessive or incorrect treatments [2].

The arachnid is nocturnal, synanthropic, and building dwelling; there is no clear correlation with direct bite frequency as the spider population increases alongside humans [2]. Painless or skin prick bites usually occur during sleep and while changing clothes when it is inconspicuously hidden and suddenly disturbed [2]. Locations include the leg, arm, and torso in most cases, and only 0.6-1.5% are on the face, neck, shoulder, buttocks, or genitalia [2]. The venom is hyaluronidase and cytotoxic sphingomyelinase-D venom mixture, promoting tissue penetration and necrosis with hemolysis, respectively, noting the “red, white, and blue sign” and risk for systemic involvement [1-3]. Through a complaint of a bite, geographical information, clinical manifestations, and laboratory evidence, the diagnosis is either putative, presumptive, probable, or documented [2]. We report a probable viscerocutaneous loxoscelism envenomation by a *Loxosceles* spider (possibly *L. apachea*, *L. blanda*, or *L. reclusa*) with lateral canthotomy in West Texas.

## Case presentation

A 44-year-old male with no past medical history presented to a freestanding emergency department (ED) reporting a spider bite to his face, just superior to the right eyebrow, that occurred while changing a ceiling fan approximately 24 hours earlier. He caught the spider, noting it to be a brown recluse, but did not bring it to the ED or take any photographs. The family also confirmed the spider bite. He denied any other initial symptoms. However, he awoke approximately eight hours later after sleeping due to facial pain, swelling, and progressive right-eye vision loss. This worsened throughout the day, ultimately prompting him to go to the ED the next evening approximately 24 hours after the initial bite.

In the ED, his triage vitals were as follows: heart rate (HR) of 129 bpm, blood pressure (BP) of 108/67 mmHg, respiratory rate (RR) of 18 breaths per minute, temperature (T) of 36.8°C, and oxygen saturation of 91% on room air. The exam was notable for a puncture mark above the right eyebrow, right-sided facial ecchymosis, swelling extending to the jawline and lips, and marked right eye proptosis. The ocular globe was poorly visualized due to severe eyelid edema with serosanguinous non-purulent discharge. Decompensation occurred within 15 minutes after the initial presentation. On the cardiac monitor, hypoxia occurred despite supplemental oxygen, and hypotension was noted with a mean arterial pressure of 30 mmHg. Diaphoresis, nausea, and worsening facial swelling ensued. Intravenous epinephrine and dopamine were administered, and intubation was completed for airway protection in the setting of clinical tracheal deviation. During intubation, tracheal deviation was confirmed via direct laryngoscopy (DL) and attributed to severe facial edema. A 7.5 Fr endotracheal tube was placed along with a left internal jugular (IJ) central venous catheter (CVC). Medications used for intubation included succinylcholine and etomidate, and a push dose of epinephrine 0.1 mg. Sedation was maintained with propofol. An orogastric tube was inserted, and the output was a mixture of 1300 mL of coffee-ground material and frank blood. As the right-sided proptosis continued to worsen while awaiting transfer to an intensive care unit (ICU), a right lateral canthotomy with cantholysis was performed for suspected ocular compartment syndrome, which resulted in the release of a significant amount of serosanguinous fluid and clinical improvement of the proptosis.

Laboratory studies were notable for a WBC count of 7.5 x 103 cells/mm3, 53% bands, and thrombocytopenia with platelets at 99 x 103/mm3. Initial hemoglobin was 10.1 gm/dL. There was metabolic acidosis with bicarbonate <10 mEq/L, an anion gap of 26.0 mEq/L, a delta gap of 14.0 mEq/L, and a delta ratio of 1.0, suggesting pure anion gap acidosis. Acute kidney injury was noted with blood urea nitrogen (BUN) at 27 mg/dL and creatinine at 3.9 μg/g. An initial serum lactate was unable to be performed due to resource constraints. Hemolysis and disseminated intravascular coagulation (DIC) were diagnosed based on the following: fibrin degradation products >20 mcg/mL, fibrinogen = 501 mg/dL, elevated D-dimer at 10.8 mcg/mL FEU, elevated international normalized ratio at 2.0, and elevated activated partial thromboplastin time (aPTT) at 62.6 seconds. A lactate dehydrogenase test was ordered with no result reported, and a haptoglobin was not ordered. The International Society on Thrombosis and Haemostasis DIC score was 6, consistent with DIC. Liver enzymes were elevated with aspartate aminotransferase at 459 units/L, alanine aminotransferase at 112 u/L, and total bilirubin at 1.1 mg/dL. Rhabdomyolysis was noted given myoglobin at 3,282 ng/mL and creatine kinase at 635 u/L.

The patient was transferred to the ICU and the regional poison control center was contacted. Dopamine and norepinephrine were maximally administered but persistent hypotension continued at 81/49 mmHg with tachycardia at 128 bpm and temperature at 99°F. His face was swollen and dark in color. Lactic acid level was 8.9 mmol/L. The medical toxicologist recommended supportive care, including steroids, blood products, reversal of any coagulation abnormalities, a bicarbonate drip, and plasmapheresis consideration. Unfortunately, the patient continued to deteriorate and not all recommended treatments were started. Nephrology was consulted for ​​continuous renal replacement therapy (CRRT) but the patient was too unstable due to profound hypotension despite four vasopressors, including epinephrine, dopamine, and dobutamine. For the profound acidosis, sodium bicarbonate 150 mEq was administered intravenously. The patient developed asystole with a lactate of greater than 12.2 mmol/L and died approximately 37 hours after the spider bite.

## Discussion

**Figure 1:**
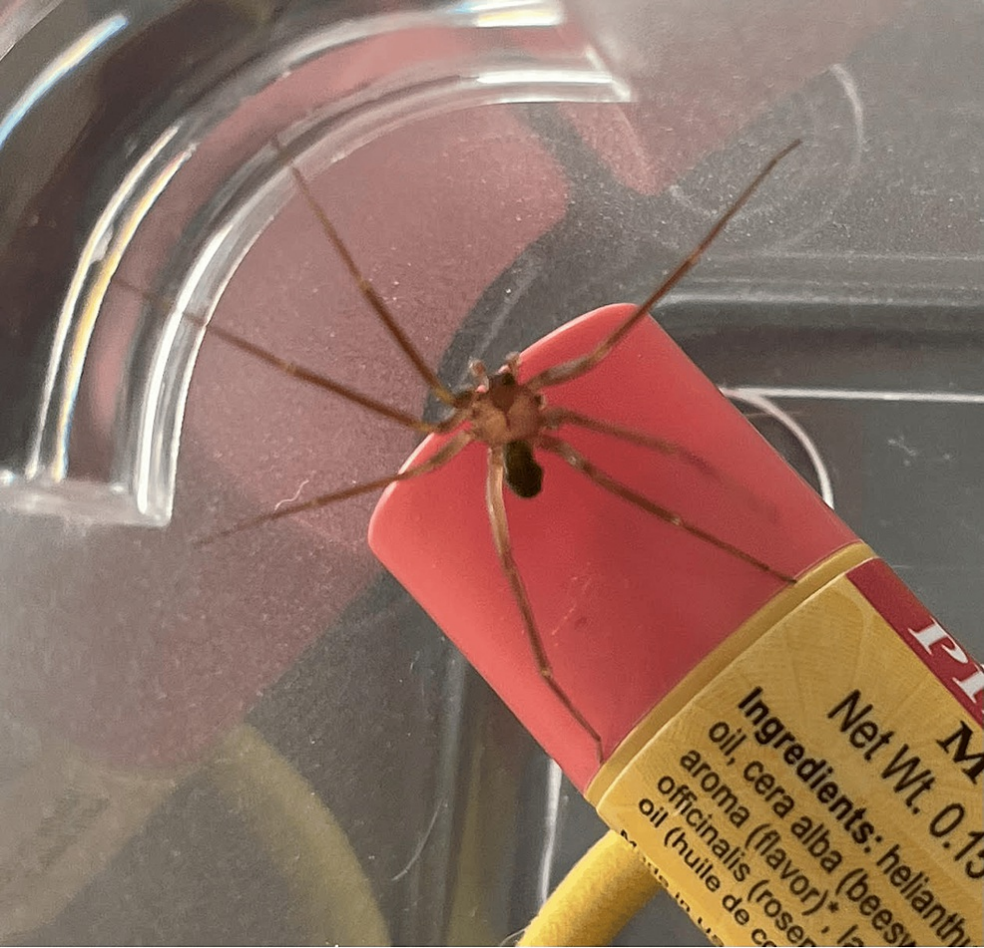
An example of a brown recluse spider. Image credits: Sarah A. Watkins.

This is a case of probable viscerocutaneous loxoscelism envenomation with subsequent DIC, acute hypoxic respiratory failure, gastric hemorrhage, and right ocular proptosis requiring lateral canthotomy with cantholysis. The differential diagnosis includes spider bites from other genera, including *Tegenaria agrestis* (hobo spider, northwestern brown spider, and Walckenaer's spider) and *Kukulcania hibernalis* (southern common house spider, most commonly misidentified) [1,3]. An example of a brown recluse spider notes the aforementioned distinguishing features (Figure 1). Other differential diagnoses were sepsis, allergic dermatitis, necrotizing fasciitis, cutaneous leishmaniasis, fungal infection, pyoderma gangrenosum, chemical burns, ischemic vascular disorders, and drug use [1,2]. The previously published mnemonic “NOT RECLUSE” can aid in clinical diagnosis, but this mnemonic needs further validation [4]. Systemic loxoscelism cases have been published previously, with three brown recluse bites involving the eyes, and one of those three involving lateral canthotomy [5-7].

Poison control recommendations were implemented except for plasmapheresis and CRRT. Fresh frozen plasma may not have been indicated (based on the prothrombin time/aPTT ratio of 0.38 and normal fibrinogen), and retrobulbar and gastrointestinal bleeding would not have supported anticoagulant use. Treatment should address cutaneous and symptomatic features simultaneously, including wound care, immobilization, tetanus prophylaxis, analgesics, antipruritics, antibiotics, and follow-up for delayed corrective surgery and chronic wound care management [2]. Equivocal evidence is demonstrated for dapsone use; colchicine, tetracycline, hyperbaric oxygen therapy, corticosteroids, and nitroglycerin have shown no benefit [2,3]. Ongoing vaccine trials and use of antivenom are noted in Latin America, with none indicated in the USA [2,4,8,9].

Previously authors have proposed a secondary warm autoimmune hemolytic anemia (AIHA) process, but the mechanism is unknown. Standard initial treatments are indicated for the ED setting with admission [10]. No Coombs test was completed, but it may be warranted in future cases.

Regarding pregnant and pediatric patients, management is similar. Data from the National Poison Data System from 2009 to 2018 showed no maternal deaths; however, three fetal deaths were reported [11]. There was a report of a pregnant patient from Mexico who had systemic features and was provided standard treatments, including dapsone and wound care [12]. A review of pediatric brown recluse pediatric complications and outcomes found 26 cases over 10 years and made similar recommendations for treatment [13]. One pediatric case report described myocarditis, pulmonary edema, and cardiogenic shock, and the patient recovered with treatment that included steroids, plasmapheresis, and intravenous immunoglobulin [14].

**Figure 2:**
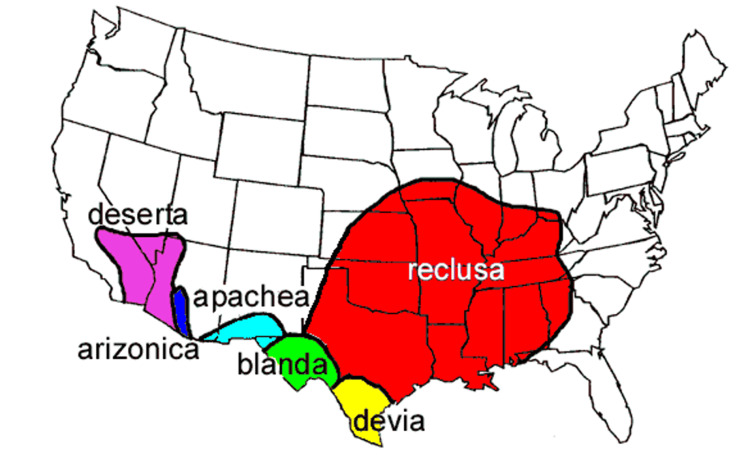
Geographic distribution of Loxosceles species. Figure adapted from [17]. Permission for use was obtained from the original publishers.

The main limitation of this case report is that we were unable to definitively determine that the patient was envenomated by a *Loxosceles* spider due to no specimen being presented to the ED clinician, photographic evidence or confirmatory testing, such as a passive hemagglutination inhibition test (PHAI) assay and toxin enzyme-linked immunosorbent assay, which were not performed due to the patient’s rapid clinical deterioration and limited resource availability [3,15]. Furthermore, limited testing was performed, such as blood cultures, due to rapid decompensation. Standard evaluation of infection and sepsis was not completed. Laboratory data were also limited and can be typical of poison control center cases. There are other case reports noting a differential diagnosis of skin infection and spider bites [16]. The temporal association of the patient’s reported spider bite, the geographic distribution of the spider, the clinical features, and the clinical deterioration are nevertheless highly suspicious for viscerocutaneous loxoscelism envenomation (Figure 2) [2,17].

## Conclusions

Systemic *Loxosceles* envenomation is a rare condition with potentially high mortality, causing rapid clinical deterioration and possible need for lifesaving surgical intervention. The acute care clinician must have a high index of suspicion for visceral manifestations in the presence of cutaneous *Loxosceles* envenomation. Systemic loxoscelism may warrant admission, interdisciplinary team coordination, and significant other healthcare resources. Future opportunities for research include standardization of laboratory work-up, such as consideration of Coombs testing, and broadening treatment options, such as antivenom therapy.
